# Combining Partial Directed Coherence and Graph Theory to Analyse Effective Brain Networks of Different Mental Tasks

**DOI:** 10.3389/fnhum.2016.00235

**Published:** 2016-05-20

**Authors:** Dengfeng Huang, Aifeng Ren, Jing Shang, Qiao Lei, Yun Zhang, Zhongliang Yin, Jun Li, Karen M. von Deneen, Liyu Huang

**Affiliations:** ^1^School of Life Science and Technology, Xidian UniversityXi'an, China; ^2^Department of Radiology, Medical Physics, University Medical Center FreiburgFreiburg, Germany; ^3^School of Electronic Engineering, Xidian UniversityXi'an, China

**Keywords:** electroencephalogram (EEG), partial directed coherence, graph theory, effective networks, mental tasks

## Abstract

**Purpose:** The aim of this study is to qualify the network properties of the brain networks between two different mental tasks (play task or rest task) in a healthy population.

**Methods and Materials:** EEG signals were recorded from 19 healthy subjects when performing different mental tasks. Partial directed coherence (PDC) analysis, based on Granger causality (GC), was used to assess the effective brain networks during the different mental tasks. Moreover, the network measures, including degree, degree distribution, local and global efficiency in delta, theta, alpha, and beta rhythms were calculated and analyzed.

**Results:** The local efficiency is higher in the beta frequency and lower in the theta frequency during play task whereas the global efficiency is higher in the theta frequency and lower in the beta frequency in the rest task.

**Significance:** This study reveals the network measures during different mental states and efficiency measures may be used as characteristic quantities for improvement in attentional performance.

## Introduction

Executive functioning (EF) is an umbrella term that refers to high level cognitive functions, which is of great importance to maintain effective goal directed behavior (Welsh and Pennington, 1988; Hughes and Graham, 2002; Alvarez and Emory, 2006). During the last decade it has become a very active research field with over 12000 scientific published articles. EF incorporates (a) the capability to inhibit or delay a particular response, (b) control the mental representation of the task with working memory, and (c) plans for action sequences (Pennington and Ozonoff, 1996; Piek et al., 2004).

One challenge in this field is the question whether executive functions are modulated by the frontal lobes (Welsh, 2002; Alvarez and Emory, 2006), producing ambiguity of definition. A mainstream view is that individuals who perform badly on executive function have “frontal lobe deficit” (Duke and Kaszniak, 2000; Stuss, 2000). However, some reserchers found that persons with frontal lesions can still perform like healthy people on executive function tests (Shallice and Burgess, 1991; Ahola et al., 1996). Moreover, persons with diffuse lesions or non-frontal lesions were even reported to perform similarly to those who had frontal lesions on these tests (Anderson et al., 1991; Axelrod et al., 1996). Furthermore, based on a meta-analytic review of previous woks, Alvarez and Emory (2006) claimed that frontal brain, as well as non-frontal brain regions, are both essential to intact executive functions which should be regarded as a more integrative process, and not merely limited to frontal brain regions.

Brain networks can be investigated with diffusion magnetic resonance imaging (DTI), functional MRI (fMRI), magnetoencephalography (MEG), and electroencephalography (EEG) (Bullmore and Sporns, 2009; Rubinov and Sporns, 2010; Lin et al., 2014). However, in contrast to the other neuroimaging techniques, EEG still remains the cheapest and most widespread technique so far. In particular, its high resolution in time domain allows the observation of signal oscillation in a scale of miliseconds.

In previous attempts to investigate the brain network organization with EEG signals, a large number of conventional methods, such as the techniques of time-domain, frequency-domain, correlation, nonlinear dynamics, and synchronism were proposed (Hjorth, 1970; Aertsen and Gerstein, 1985; Sandkühler and Bhattacharya, 2008; Knyazeva et al., 2010). These methods clarified the network features in the analysis of directionless functional connectivity. However, they were not able to provide direct insights into the directional flows of information among network nodes. In fact, the human brain is a complex dynamic system and constantly responds to the external stimuli (Fox et al., 2005). Thus, it is crucial to explore the directional information flows of the instantaneous interactions of the brain among several nodes.

Previous studies have provided several kinds of sophisticated EEG analysis techniques to study the directional interactions between any pair of network nodes or cortical areas (Baccalá and Sameshima, 2001; Kamiński et al., 2001; Schelter et al., 2006; Supp et al., 2007; De Vico Fallani et al., 2010, 2007). For example, partial directed coherence (PDC) on the basis of the Granger causality (GC) (Granger, 1969) and the multivariate autoregressive (MVAR) model (Blinowska et al., 2004) has attracted more attention since it can be adopted to characterize the effective connections, representing direct or indirect causal influences of one region to another (Rubinov and Sporns, 2010). Using graph theoretical approach (Sporns et al., 2004; Bullmore and Sporns, 2009; Van Dellen et al., 2009), topological properties of complex networks were characterized (Ahmadlou et al., 2012; Zhang et al., 2013, 2015; Gast et al., 2014; Gupta and Falk, 2015; Liu et al., 2015; Youssofzadeh et al., 2016).

The experiment in this study was designed to investigate the attention aspect of the effective function. The aim of the current work is to investigate the brain networks using electrodes related to different mental tasks in a healthy population. Moreover, the question is whether the effective network, evaluated by graph analysis, may be different based on the above chosen electrodes. In particular, the main experimental questions we would like to know from the study are the following:

1. Is the efficiency index significantly different in the obtained brain networks in different mental states?
2. If it does exist, is such a difference related to a specific frequency?

To the best of our knowledge, this is the first study that combined PDC and graph theory to evaluate the effective networks properties toward different mental states in a healthy population. In particular, the efficiency indexes with regard to the four EEG rhythms (delta, theta, alpha, and beta) were closely investigated. Moreover, it shed light on the brain network toward executive functions in a healthy population.

## Methods and materials

### Subjects

The nineteen university students participating in the study were recruited from Xidian University (mean age ±*SD* = 24.25 ± 1.57 years; right-handed; male; visual acuity or corrected visual acuity no less than 1.0). They had a full-scale Raven's Standard Progressive Matrices score of 100 or above. Written, informed consent was obtained from each subject after the explanation of the study, which was approved by the Ethical committee of the institute of life science and technology of Xidian University. The subjects had no personal history of neurological disease, clinically significant head trauma, a substance use disorder, or psychiatric illness. The participants were not allowed to take any medication, coffee, and alcohol for a minimum of 4 h before the experiment.

### Experimental design

Two mental tasks have been designed for participants as follows (Yan et al., 2007):

- Play task: participants were comfortably seated on a sofa in front of a computer, about 72 cm from the screen. Each subject was asked to focus the attention on a game called “feeding frenzy.” The participants had to use the mouse to control “their fish.” The controlled fish swam around the sea, adding points by eating smaller fish but avoiding bigger fish simultaneously. One session lasted for 3 min. To ensure the quality of the data, another session was recorded.
- Rest task: all the surroundings and requirements were the same as in the play task. The only difference was that the subjects were asked to sit in silence while watching the still image of the game on the screen and should not think about anything special. One session lasted for 3 min. Two sessions were recorded.

### EEG acquisition

EEG data were recorded at 16 scalp loci (FP1, FP2, F3, F4, C3, C4, P3, P4, O1, O2, F7, F8, T3, T4, T5, T6, and A1+A2 as reference) in line with the international 10–20 system to linked earlobes. The experiments were performed in a normal chamber without noise interference. The electrodes and the positions connecting with the electrodes were defatted with alcohol to reduce the impedance and interference. Impedance was kept below 10 *k*Ω. EEG signals of each subject were recorded with a 32-channel EEG system (NT9200, Beijing, China) when performing different mental tasks. The signals were recorded with a bandpass of 0.05–30 Hz, 50 Hz notch, and digitized at 1000 Hz.

### Pre-processing

**Figure 1:**
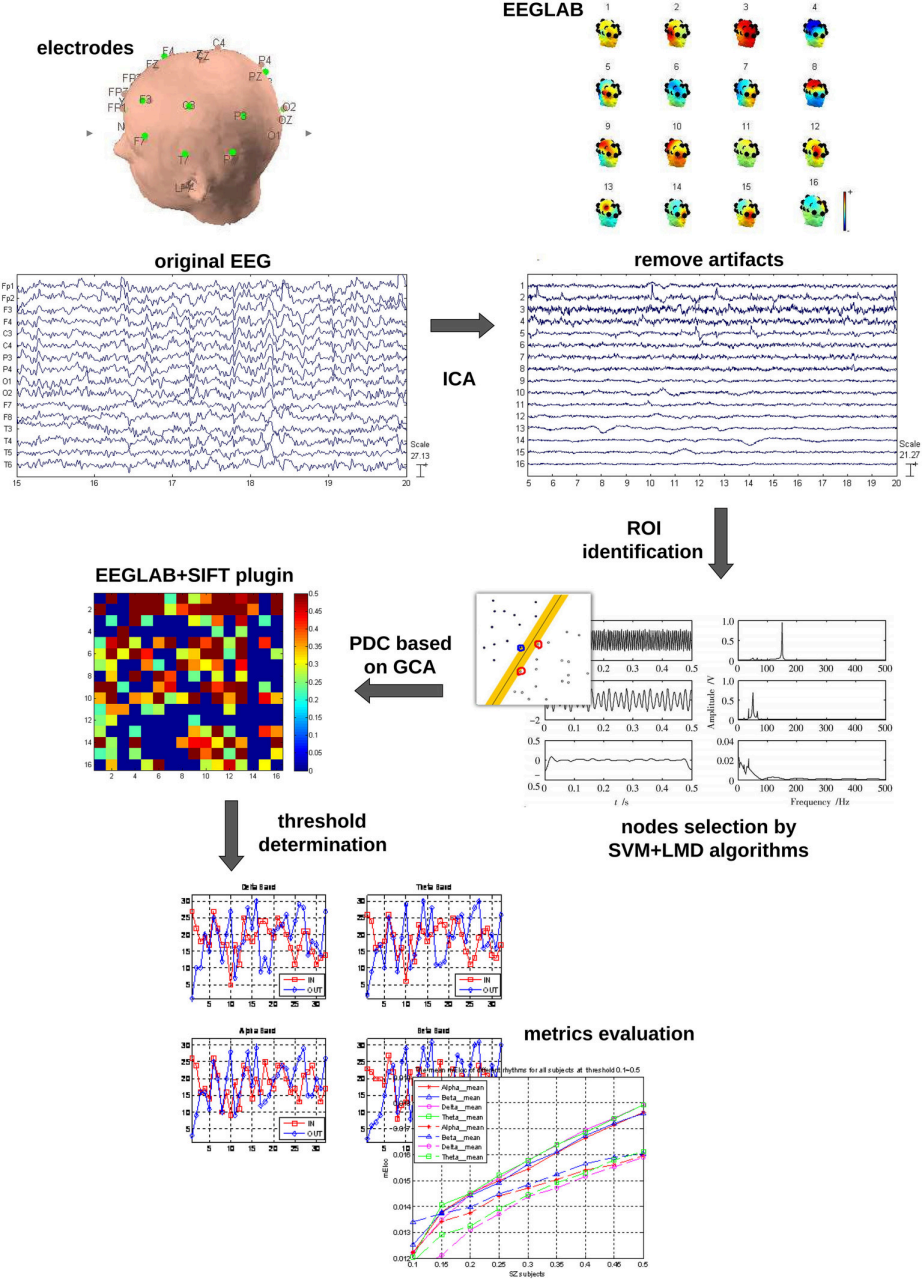
**Data processing workflow**.

The whole data processing work flow is summarized in Figure 1. The pre-processing included: (1) The EEG data were down-sampled to 250 Hz to reduce the volume of the data. (2) The frequent swallowing and winking fragments of the EEG data were diminished by means of independent component analysis (ICA) employing the function runica from EEGLAB (Delorme and Makeig, 2004).

### Electrodes selection by LMD and SVM

Some investigators suggested that (Pardo et al., 1991; Buschman and Miller, 2007) the activation of frontal and parietal cortical areas are involved in the process of attention. However, it is not sure yet how many of the electrodes should be accurately included. To reduce the number of electrodes for future clinical use and eliminate a source of subjectivity, in this work, the technique combining LMD and SVM was used to classify the two mental tasks for each subject.

LMD (Smith, 2005; Park et al., 2011) is an iterative method to demodulate amplitude and frequency modulated signals. It decomposes the EEG signal into a set of components or functions. Each of the components is the product of an envelope signal and a frequency modulated signal. In our study, the energy of the first four components was summed as the input vector for the SVM (Burges, 1998; Noble, 2006; Hsu et al., 2008). Our SVM model was developed in LIBSVM (version 3.1) with Radial basis function (RBF) kernel parameters. To choose the optimal parameters for gamma-g and cost-c, which affected the performance of SVM model greatly, the genetic algorithm (GA) was used for every data set in a cross validation manner. In particular, leave-one-out cross validation was used in our study to ensure the results reliable and repeatable.

The results of SVM classification for all the EEG electrodes (Table 1) show that different EEG electrodes result in different classification precisions, and 10 electrodes (F3, F4, C3, C4, P3, P4, T3, T4, T5, and T6) with classification precision higher than 60% were chosen as the network nodes.

**Table 1:** **A comparison of classification precisions among various EEG electrodes**.

**Electrodes**	**Precision (%)**	**Electrodes**	**Precision (%)**
Fp1	47.36	O1	55.26
Fp2	44.73	O2	52.63
F3	76.31	F7	57.89
F4	63.15	F8	57.89
C3	81.57	T3	68.42
C4	65.78	T4	60.52
P3	77.89	T5	73.68
P4	69.26	T6	60.52

### Partial directed coherence (PDC)

The concept of PDC, proposed by Baccala and Sameshima in 2001 (Baccalá and Sameshima, 2001), put forward a new frequency-domain method for the description of GC.

Generally, a MVAR model with *m* channels of EEG signals and order *p* is defined by,

(1) $$\begin{array}{r} \text{X}\left(\text{t}\right)=\sum_{r=1}^{p}A\left(r\right)X\left(t-r\right)+E\left(t\right) \end{array}$$

Where X(t) is the vector of *m* channels of EEG signals at time *t* and the matrix *A*(*r*) contains the *r*^th^ order AR parameters. *E*(*t*) denotes the estimated error, which is supposed to be an uncorrelated Gaussian process with zero mean. The fitting results of MVAR model will be influenced by the model order *p* and Akaike information criterion (AIC) can be used to determine the proper model order *p* for our study.

Once the coefficients of the MVAR model were adequately estimated, *A*(*f*) can be obtained by $A\left(f\right)=\overset{p}{\underset{r=1}{\sum }}A\left(r\right)e^{-i2\pi fr}$. Further, the transfer function of the *m* channels of EEG signals can be quantified by $Ā\left(f\right)=I-A\left(f\right)=\left[\bar{a_{1}}\left(f\right)\bar{a_{2}}\left(f\right)\ldots \bar{a_{m}}\left(f\right)\right]$, where the elements of *A*(*f*) are defined as:

(2) $$\begin{array}{l} {\bar{A}}_{ij}\left(f\right)=\{\begin{array}{c} 1-\overset{p}{\underset{r=1}{\sum }}a_{ij}\left(r\right)e^{-i2\pi fr},\;if\;i=j\\ -\overset{p}{\underset{r=1}{\sum }}a_{ij}\left(r\right)e^{-i2\pi fr},\;otherwise \end{array} \end{array}$$

Finally, the PDC from channel *j* to *i* can be given by,

(3) $$\begin{array}{l} PDC_{ij}\left(f\right)=\frac{{\bar{A}}_{ij}\left(f\right)}{\sqrt{{\bar{a}}_{j}^{H}\left(f\right){\bar{a}}_{j}\left(f\right)}} \end{array}$$

where ā_*i*_(*f*)(*i* = 1, 2, …*M*) is the *i*^th^ column of the matrix Ā(*f*) and *PDC*_*ij*_ denotes the direction and intensity of the information flow from channel *j* to *i* at the frequency of *f*.

Given that the sliding time window techniques might disclose higher resolution dynamics (Ding et al., 2000), a sliding time window of 2 s of EEG data with 50% overlapped was used in our study. The EEGLAB toolbox, which provides functions to calculate AIC (Cui et al., 2008; Janacek, 2010), was used to determine the proper order of the MVAR model for each subject. Furthermore, a bootstrap method (Kamiński et al., 2001) was used to determine the statistical significance regarding to the above interactive networks.

### Creating adjacency matrices

So far, the connectivity matrices have been created with values of PDC between all pairwise associations for each EEG sub-band (delta, theta, alpha, and beta) in different mental states. Further, a threshold was applied to convert the connectivity matrices to adjacency matrices.

The choice of threshold is crucial in the process of generating an adjacency matrix from the association matrix. Conceivably, different thresholds will result in graphs of different connection densities or sparsities. Although there are currently several techniques able to select the threshold, e.g., surrogate-data technique (Pritchard et al., 2014), significance level based technique (De Vico Fallani et al., 2007), and so on, there is currently no consent on which one is optimal.

Here, a method based on significance level was employed to determine the threshold *T* in this study. The effective network was converted into a directed unweighted graph and the connection matrix containing PDC values for each directed pair of nodes into an adjacency matrix **A** by means of a threshold *T*. The threshold *T* can be viewed as the ratio of the number of real effective connections to the number of all possible connections in this network, and *T* indicates the number of the most powerful interactions to be considered. In order to explore the features of the effective networks at different connection densities, the threshold *T* was slid within the interval 0.1–0.9 with a step of 0.05 and the average values of local efficiency in beta rhythm were obtained under the related threshold between the two mental states.

### Graph analysis

In this paper, degree, degree distribution and efficiency indexes were calculated with the Brain Connectivity Toolbox (Rubinov and Sporns, 2010) under Matlab R2010a. In particular, efficiency is a quantity that measures how efficiently the information is exchanged over the network (Latora and Marchiori, 2001; Fan et al., 2002; Goulas et al., 2015). The efficiency *e*_*ij*_ in the communication between vertices *i* and *j* is defined as *e*_*ij*_ = 1∕*d*_*ij*_ ∀*i, j*, where *d*_*ij*_ is the shortest distance between the nodes *i* and *j*. If there is no path in the graph between the nodes *i* and *j* then *d*_*ij*_ = ∞, and therefore *e*_*ij*_ = 0.

The global efficiency of **A** is defined as: $E_{\text{glob}}=\frac{1}{N\left(N-1\right)}\underset{i\neq j}{\sum }\frac{1}{d_{i,j}}$, where is the number of vertices in the graph. The local properties of **A** can be characterized by evaluating for each node *i* the efficiency of **A**_*i*_ (the sub-graph of the neighbors of *i*). The local efficiency is the average efficiency of the local sub-graphs, i.e., $E_{\text{loc}}=\frac{1}{N}\sum_{i=1}^{N}E_{\text{glob}}\left({\text{A}}_{i}\right)$. This property tells us the “fault-tolerant” of the network system, to be more specific, how efficient is the communication between the first neighbors of *i* when *i* is removed (De Vico Fallani et al., 2007; Klados et al., 2012, 2013).

## Results

In the study, the optimal model order for each subject obtained from AIC was in the range between 10 and 16. The connectivity matrixes were created with the values of PDC between all pairwise electrodes for each EEG sub-band following a subject specific MVAR order in different mental states.

**Figure 2:**
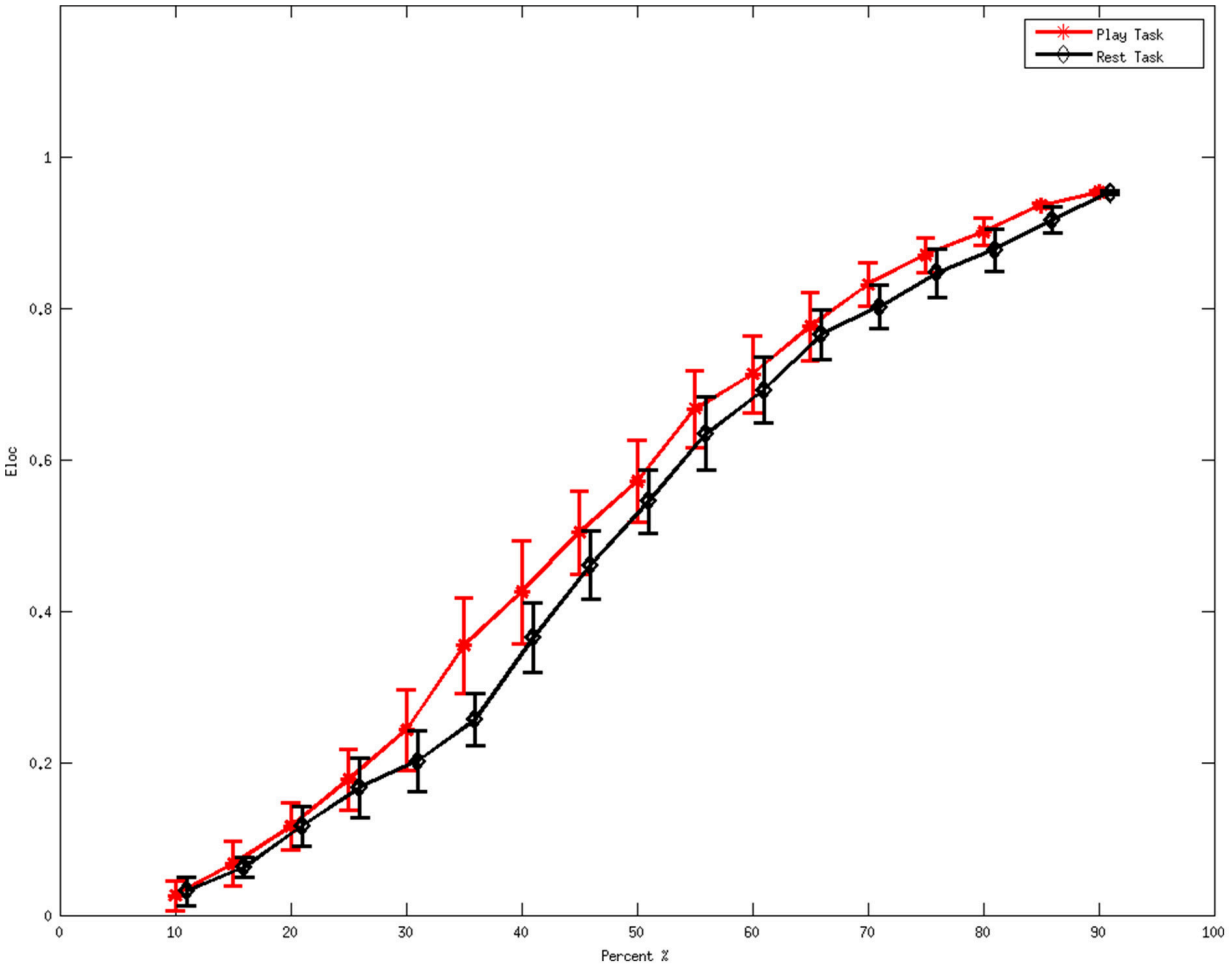
**Average values of local efficiency at various values of the threshold during the mental tasks for the beta rhythm**. The red line refers to the values in the play task and the black line represents the rest task. The short vertical lines denote the 95% confidence intervals for each threshold in the interval 0.1 to 0.9 with a step of 0.05.

Figure 2 denotes the average values of the local efficiency in the two analyzed mental states in the beta rhythm at different thresholds. Red line represents values for the local efficiency in the subjects during the play task whereas black line refers to the local efficiency during the rest task. The short vertical lines denote the 95% confidence intervals for each threshold in the interval 0.1–0.9 with a step of 0.05. Within the sub-interval of threshold in which efficiency remains significantly different (*p* < 0.05) between the two mental states, 0.35 was used to illustrate the following results.

### Degrees and degree distributions

**Figure 3:**
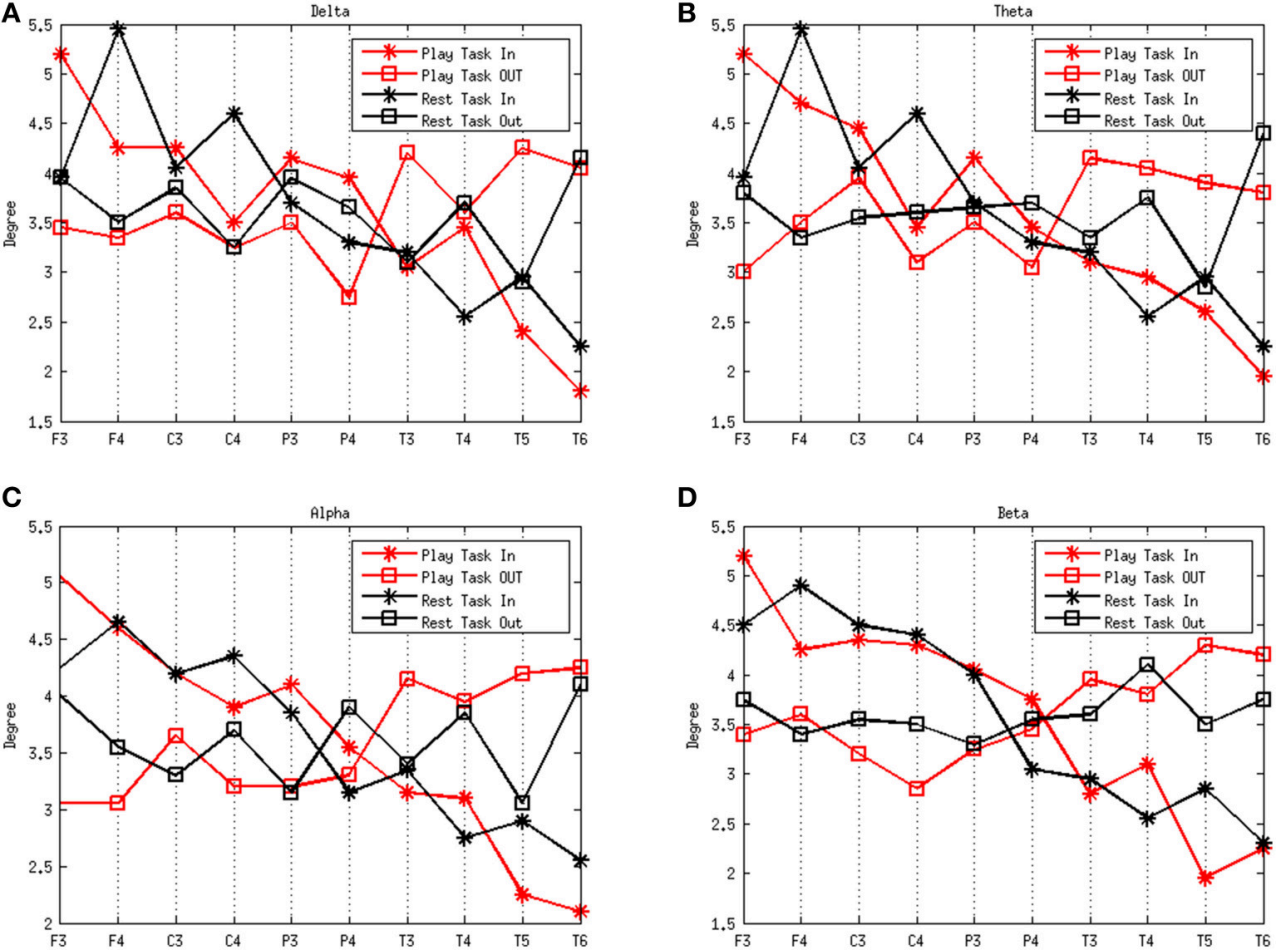
**Average degrees of the play and rest task in the four rhythms delta (A), theta (B), alpha (C), and beta (D)**. The red lines represent the play task and the black lines refer to the rest task. The stars represent the degree in and the squares refer to the degree out. The *x*-axes represent the network nodes and the *y*-axes refer to the average degree of all the subjects for each network node.

Figure 3 shows the degrees of the subjects in the frequency rhythms analyzed during different mental states. The delta, theta, alpha, and beta rhythms during the play task and rest task are shown in Figures 3A–D, respectively. A high degree-in denotes that a region is more likely to be influenced by a number of other brain regions whereas a high degree-out suggests a large quantity of functional targets. The results show that the degree-in follows an overall downward trend along the electrodes F3, F4, C3, C4, P3, P4, T3, T4, T5, and T6 in both mental states. However, notable flows outgoing (efferent) from the left temporal region (T3) are present in the play task, and flows outgoing from the right temporal region (T4) exist in the rest task. On the other hand, notable flows incoming (afferent) to left frontal region (F3) are present in the play task, and flows incoming to the right frontal region (F4) exist in the rest task.

**Figure 4:**
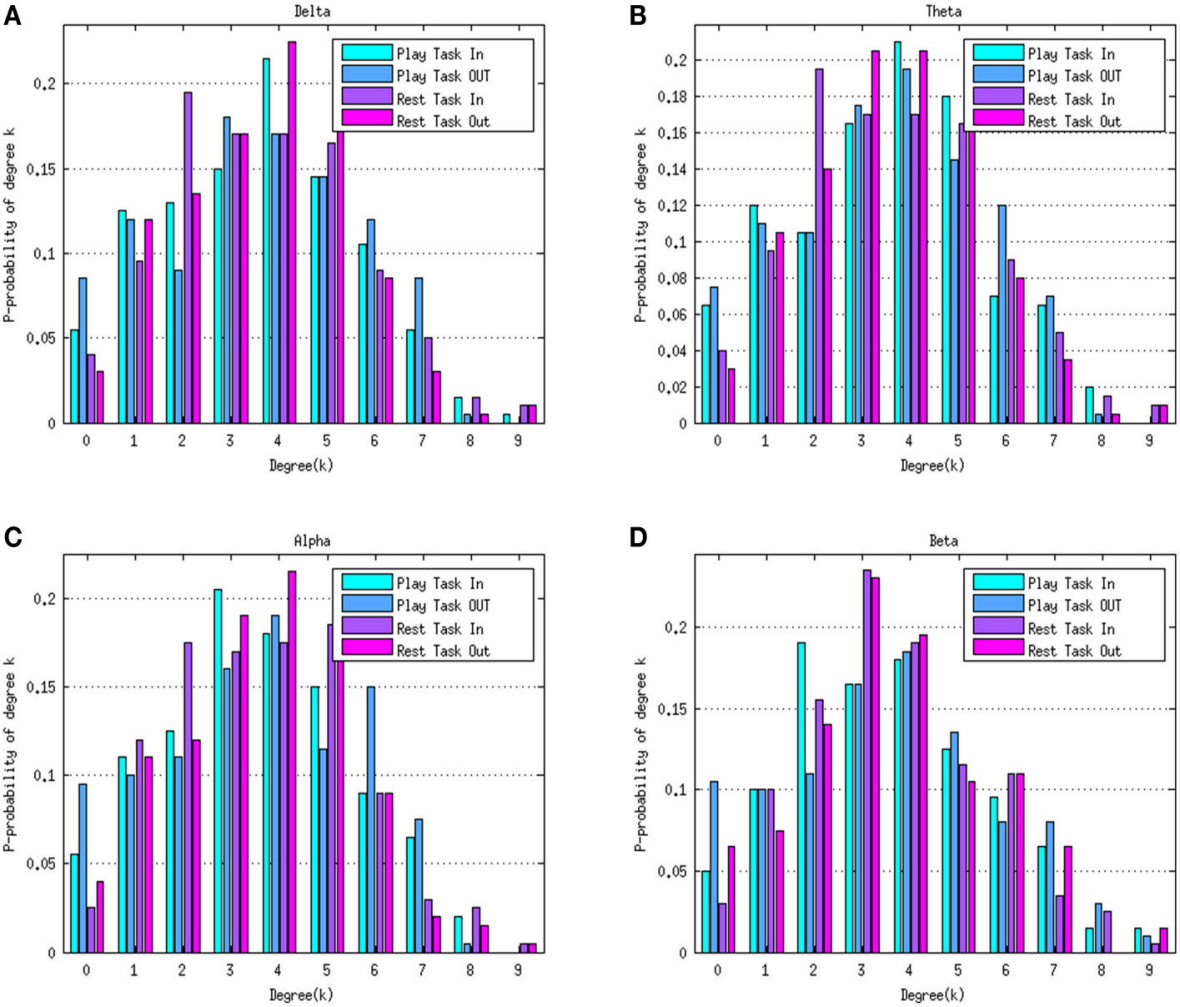
**Degree distributions of the play task and rest task in four rhythms delta (A), theta (B), alpha (C), and beta (D)**. Green bars represent the in-degree distribution and blue bars refer to the out-degree distribution in the play task. The purple bars represent the in-degree distribution in the rest task and the pink bars represent the out-degree distribution in the rest task. Histogram values were normalized to the number of the elements within the network (10 nodes).

Figure 4 depicts the average degree distributions for the two mental tasks in various rhythms. The delta, theta, alpha, and beta rhythms during the play task and rest task are shown in Figures 4A–D, respectively. In order to compare the results, the normalized histogram values were taken into consideration. Right-skew tail of in-degree distributions in the play task in beta frequency indicates the presence of few nodes with a very high level of out-going connections.

### Local efficiency and global efficiency

To investigate the inter-individual variance of the results, the local and global efficiency were calculated for the networks estimated from each volunteer during the two mental states (play and rest) in the four rhythms (delta, theta, alpha, and beta). Furthermore, analysis of variance (ANOVA) was performed on the efficiency indexes to investigate the relationships between efficiency indexes, frequency bands, and different mental states.

**Figure 5:**
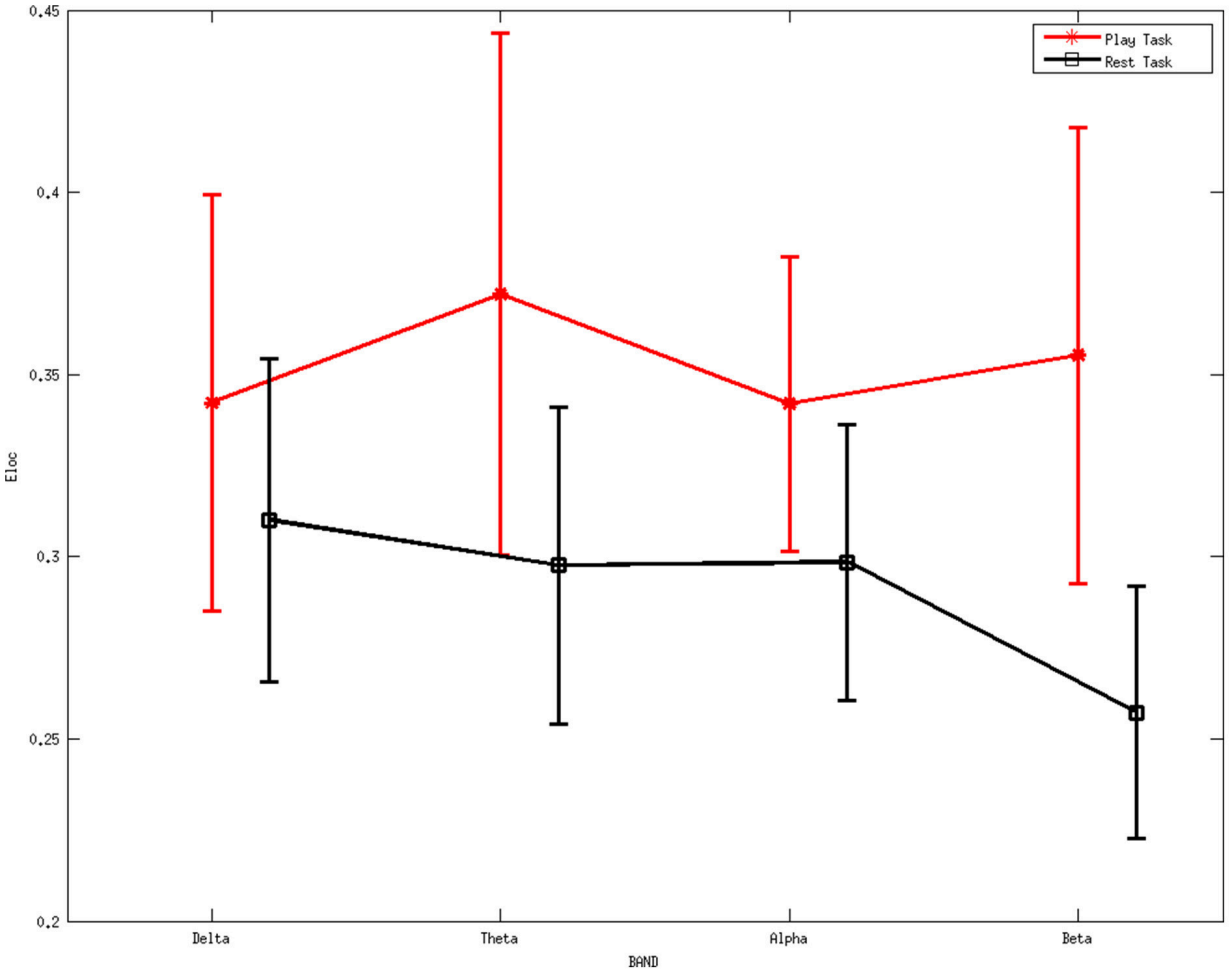
**Average values of local efficiency for various rhythms between two mental tasks**. A statistically significant difference (*p* < 0.05) was noted between them. Red line represents the average values of the local efficiency during the play task, black line refers to the rest task, while vertical bars denote 95% confidence intervals.

Figure 5 shows the average local efficiency for various rhythms between two mental tasks. A statistically significant difference (*p* < 0.05) was noted between the two mental states. Red line represents the average values of the local efficiency during the play task, black line refers to the rest task, while vertical bars denote 95% confidence intervals.

ANOVA was further performed on the local efficiency. It showed no significant differences for the factor frequency band (*F* = 0.46, *p* = 0.7105) but significant differences for the factor group (*F* = 13.4, *p* = 0.0003). The interaction between group and band were found to be not significant (*F* = 0.77, *p* = 0.5131).

**Figure 6:**
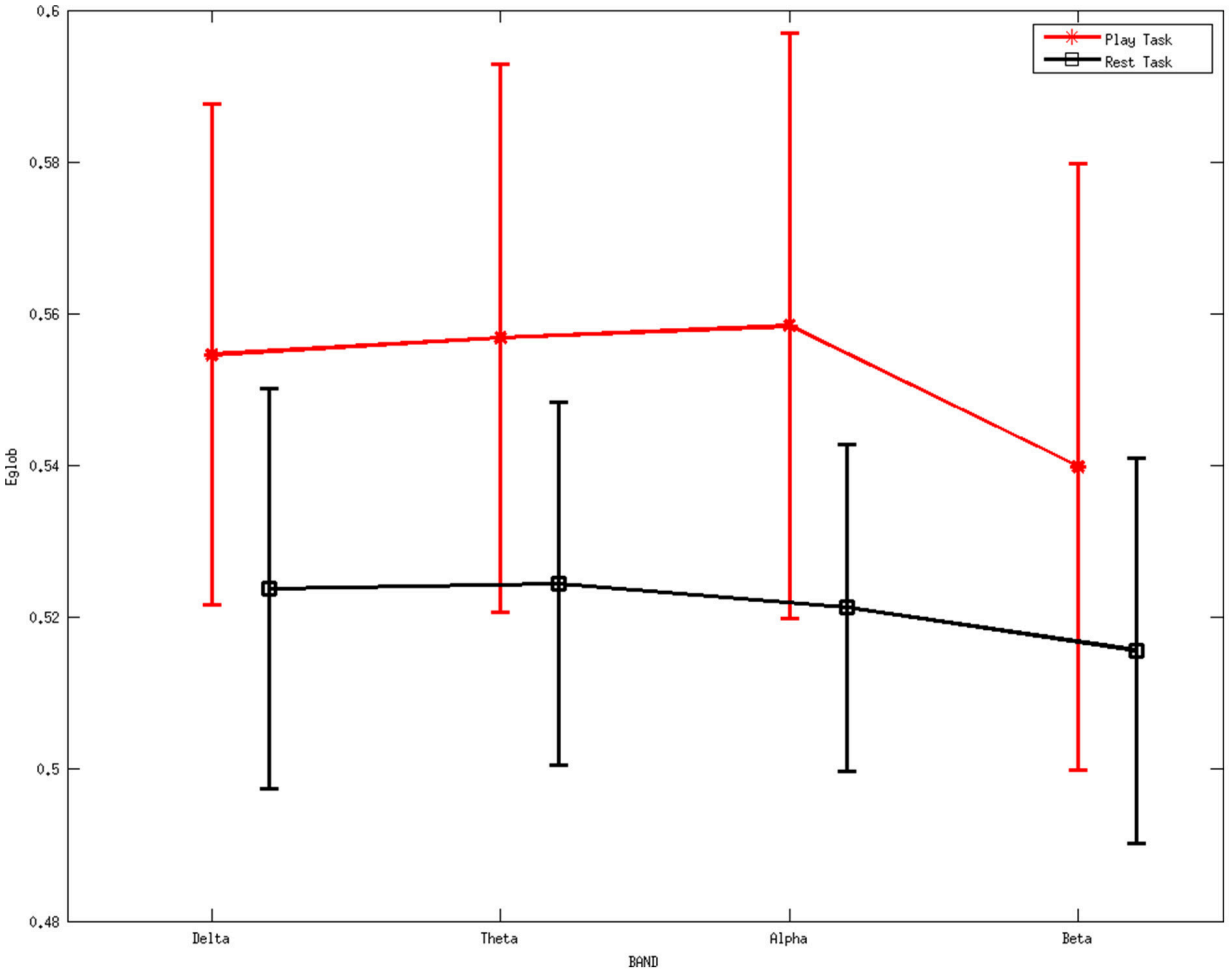
**Average values of global efficiency for various rhythms during the two mental tasks**. A statistically significant difference (*p* < 0.05) was noted between them. Red line represents the average values of the local efficiency during the play task, black line refers to the rest task, while vertical bars denote 95% confidence intervals.

Figure 6 describes the average global efficiency for various rhythms between two mental tasks. There is a statistically significant difference (*p* < 0.05) between the two mental states.

ANOVA was also performed on the global efficiency. The results showed no differences for the factor frequency band (*F* = 0.32, *p* = 0.8097) but significant differences for the factor group (*F* = 8.93, *p* = 0.0033). Moreover, the interaction between group and band were found to be not significant (*F* = 0.06, *p* = 0.9789).

## Discussion

### Assessment of electrodes selection

Using the technique combining the LMD and SVM, we quantitatively selected the electrodes most related to different tasks. Results demonstrated that 10 electrodes (F3, F4, C3, C4, P3, P4, T3, T4, T5, and T6) had a predicted accuracy higher than 60%, therefore, we consider that the information from these electrodes can reflect the different mental states better than the other electrodes.

Generally, the first step in the construction of a structural or functional brain network is the definition of network nodes (Bullmore and Sporns, 2009). These can be defined either from EEG electrodes or cortical areas with a head model or anatomically selected regions from histological data, MRI or DTI (Bullmore and Sporns, 2009; Rubinov and Sporns, 2010). For our costumed EEG experiment, the data driven method combining LMD and SVM was used to quantitatively select the proper EEG electrodes. LMD proposed by Smith (2005) has been applied in a set of scalp EEG visual perception data. The idea that it can decompose signals into a series of components or functions characterized by the frequency seems interesting for EEGs, since it is widely accepted that different frequencies of EEGs (gamma, alpha, theta, and delta) are related to certain aspects of cognitive function.

SVM has been extensively used recently in classification in EEG (Hosseini and Khalilzadeh, 2010; Subasi and Gursoy, 2010; Temko et al., 2011; Li et al., 2013; Wang et al., 2014; Hosseini et al., 2015) and fMRI (LaConte et al., 2005; Mourão-Miranda et al., 2005; De Martino et al., 2007; Altmann et al., 2015). Given that the performance of SVM depends on the choice of gamma-*g* and cost-*c*, the GA was conducted on every dataset in a cross validation manner in order to select the optimal parameters for the SVM model. In this way, one can get the highest predicted accuracy as well as smallest c, since a higher c may contribute to “overfitting.” More precisely, the leave-one-out cross validation was used to ensure that the results from the experiments can be reproducible and reliable.

The technique combining LMD and SVM resulted in 10 chosen electrodes (F3, F4, C3, C4, P3, P4, T3, T4, T5, and T6). According to the layout of international 10–20 system, F3 and F4 electrodes are located in the frontal lobe. As is well know that, frontal lobe associates with attention, short-term memory, motivation, and planning (Stuss and Knight, 2002). Thus, the described electrodes make sense from the physiological point of view. C3 and C4 electrodes were located on the edge of the frontal lobe, so that these two electrodes should be included too. Furthermore, in our play task, the subject had to control the move of the “fish” in the game by a mouse, therefore, it was not unexpected that P3 and P4 electrodes showed a good classification: These electrodes were located in the parietal lobe, which is known for sensory information among various modalities, including the sense of touch in the somatosensory cortex (Fogassi and Luppino, 2005). Moreover, T3, T4, T5, and T6, which were located in the temporal or posterior temporal lobe, showed a high distinction among the two different mental states probably because in the play task, one had to open the eyes and concentrate on the game; on the other hand the temporal lobe is related to the process visual memories into interpretations.

The technique of combining LMD and SVM in this study was applied in the data from each subject and 10 electrodes were defined as the nodes of the brain network. In this way, a major source of subjectivity was eliminated and no prior knowledge was required to define the ROI.

### Effective connectivity in different mental states

In this work, PDC and graph theory were applied to study the effective brain networks in normal subjects during different mental tasks. Combining SVM with LMD, we identified the network nodes. The distinctive properties of the effective networks were adequately analyzed from the perspective of the degree, degree distribution, as well as local and global efficiency. As mentioned above, the combination of these techniques improves the capability of detecting relevant features of the effective brain networks.

From the analysis performed on the effective network from a healthy population in different mental states, we found that nodes in the frontal lobe (F3 and F4) in both mental states showed overall the highest in-degree. It suggests that there is a great influence from other regions of the brain. This means, the brain regions around these electrodes may act as “hub” for the in-flow of information in different mental states. Removal of this region from the estimated network is more likely to cause a collapsing of the whole system. We also demonstrated notable flows outgoing from the left temporal region (T3) in the play task, and flows ingoing from the right temporal region (T4) in the rest task. Moreover, notable flows ingoing to left the frontal region (F3) exists in the play task, and flows ingoing to the frontal region (F4) exist in the rest task. The phenomenon above may be explained by the division of the left and right hemispheres. More specifically, the left hemisphere takes more responsibility for logical thinking, such as planning a best way to escape from dangerous situation in the play task.

Although the average trend of degree distributions for the two mental tasks in various rhythms (Figure 4) seems a “scale free” property, it is difficult to formally assert this conclusion, because the size of the networks estimated in the study is not big enough to achieve a reliable degree distribution.

The higher values of local efficiency during the play task (Figure 5) result from the involvement related to the execution of “feeding frenzy,” which may imply that the networks have a larger level of the internal organization and fault tolerance (Pfurtscheller and Lopes da Silva, 1999; Sivan et al., 1999; Crucitti et al., 2003). The downward trend of local efficiency from the delta rhythm to the beta rhythm in the rest task indicates a more intense mental activity in the other rhythms than the beta rhythms. The properties above may suggest a more concentrated attentiveness as a local response in the play task. Furthermore, the higher global efficiency indexes in the play task suggest stronger information transfer ability compared with the rest mental states.

### Methodological consideration

Another method toward evaluating causal relations in neural system is directed transfer function (DTF) (Kamiński et al., 2001). It also estimates the parameters for the MVAR models which derive from the multi-channel EEG signals and then transformed into the frequency domain. It has been successfully applied in several actual neurobiological recordings (De Vico Fallani et al., 2007; Liu et al., 2010; Nan et al., 2010; Dissanayaka et al., 2015). In particular, the combination of DTF with short window AMVAR method (Ding et al., 2000) is able to get the dynamic causal relations among cortical regions or EEG electrodes. However, in the simulation (not shown here), when the value of DTF between two interactions is not zero, it does not mean that there is a causal relationship between the two interactions (Baccalá and Sameshima, 2001). In other words, DTF is prone to be affected by possible alternative interactions or unpredictable factors. On the other hand, PDC which has been widely used recently (Schelter et al., 2006; Supp et al., 2007; Michels et al., 2013; Silchenko et al., 2013; Pester et al., 2015; Youssofzadeh et al., 2016), illustrates the relative strength of direct interactions and avoids the inverse operation, therefore reducing the computation time. With this consideration, PDC is more reliable and faster to quantify the causal interactions among multi-channel EEG signals in our study.

### Limitation and future implications

We have to mention that the predicted accuracies from electrodes (Table 1) were overall not high. It may due to two factors. First, the experiments were carried out in a small chamber without electrical interference. We have mentioned that the experiment is part of the “personalized neurofeedback treatment and brain mechanisms project.” We therefore tried to set all the conditions similar to the home environment for the further comparison between a healthy population and patients. The second reason is that we placed the position of each electrode one by one manually according to layout of international 10–20 system; therefore, individual difference may exist between different experiment operators.

There are several implications of the present study. Firstly, the participants in our study were university students. Previous studies showed that the executive function is related to the neurobiological development of the brain (De Luca and Leventer, 2008). Furthermore, Liu et al. (Liu et al., 2015) has reported the properties of attention-related functional networks between ADHD and normal control children with EEG. Therefore, it would be interesting to find how the efficiency indexes are for ADHD Children from the information flow perspective. However, this is not the scope of the present study.

Secondly, the threshold we used in our study was based on the paired *t*-test according to the local efficiency during different mental states in the beta frequency, which was considered to be the most prominent band in our experiment. This threshold could destroy the cohesion of the network. Since there is currently no consent on how to choose an optimal threshold, we would like to skip this step by analysing weighted graph directly in the future study.

What is more, the fact that the local efficiency is higher in the beta frequency and lower in the theta frequency during play task while the global efficiency is higher in the theta frequency and lower in the beta frequency in the rest task suggests that these network properties could be indexes to classify different mental states in a healthy population. Similarly, Ahmadlou et al. (2012) showed that the short path lengths and clustering coefficient can distinguish the ADHD brain from non-ADHD from the delta EEG sub-band. Petti et al. (Petti et al., 2013) used the small-worldness to classify the populations between young and mid-aged adults with EEG.

## Conclusions

This study aimed to investigate the effective networks in a healthy population in different mental states without the prior knowledge for the region of interest for the experiment design. We have demonstrated that the graph theory combined with the PDC can describe the local and the global properties in the effective connections estimated from the EEG signals. The network measures, such as degrees together with their distributions, local and global efficiency, were applied to investigate the properties of the effective network between the different mental tasks.

The present study can be also regarded as the step of investigation of executive function of attention, to which eventually aim at investigation from ADHD population in the future. It is expected that our study will have useful and valuable implications for preclinical studies.

## Author contributions

Conceived and designed the experiments: DH, AR, and QL. Performed the experiments: YZ and ZY. Analyzed the data: DH and SJ. Contributed reagents/materials/analysis tools: KV. Wrote the paper: DH and LH.

### Conflict of interest statement

The authors declare that the research was conducted in the absence of any commercial or financial relationships that could be construed as a potential conflict of interest.
